# Derangement of protein S and C4b-binding protein levels as acquired thrombophilia in HIV-infected adult Nigerians

**DOI:** 10.4102/sajhivmed.v22i1.1253

**Published:** 2021-08-23

**Authors:** Fatai O. Bello, Alani S. Akanmu, Titilope A. Adeyemo, Bukunmi M. Idowu, Prosper Okonkwo, Phyllis J. Kanki

**Affiliations:** 1 Department of Haematology and Blood Transfusion, Lagos University Teaching Hospital, Idi-Araba, Lagos State, Nigeria; 2 Department of Radiology, Union Diagnostics and Clinical Services, Yaba, Lagos State, Nigeria; 3 APIN Public Health Initiatives, Abuja, Nigeria; 4 Harvard School of Public Health, Boston, Massachusetts, United States of America

**Keywords:** HIV, thrombosis, protein S deficiency, protein C deficiency, C4b-binding protein, clot lysis, euglobulin clot lysis time, tissue plasminogen activator, fibrinolysis

## Abstract

**Background:**

HIV is a chronic inflammatory state with the production of many acute-phase-reactant proteins. Some of these proteins have procoagulant activities that predispose HIV-infected patients to thrombosis.

**Objectives:**

The aim of the study was to evaluate the effects of HIV infection on the serum levels of C4b-binding protein (C4BP) and protein S as markers of predisposition to thrombosis in HIV-infected adults.

**Methods:**

The study population comprised of 61 HIV-infected adults on antiretroviral treatment (ART) who had achieved virological suppression, 58 HIV-infected adults not yet on ART and 59 HIV-negative healthy controls. The serum levels of free protein S, C4BP and the euglobulin clot lysis time (ECLT) were determined.

**Results:**

The mean plasma-free protein S level of HIV-infected patients on ART (86.9% ± 25.8%) was significantly higher than that of treatment-naïve HIV-infected patients (75.7% ± 27.3%) (*p* = 0.005). Conversely, there was no statistically significant difference between the protein S levels of the HIV-infected subjects on ART (86.9% ± 25.8%) and those of the controls (94.9% ± 7.9%) (*p* = 0.119). The mean C4BP was significantly higher in the treatment-naïve HIV-infected subjects (36.7 ± 1.7 ng/dL) than that in those on ART (30.7 ± 2.6 ng/dL) and that in the controls (22.4 ± 2.4 ng/dL) (*p* < 0.0001). Protein S deficiency was more prevalent among the subjects with elevated C4BP (*p* = 0.023). The mean ECLT was significantly more prolonged in the treatment-naïve HIV-infected subjects (241.9 ± 34.7 s) than controls (189.5 ± 40.7 s) (p < 0.0001).

**Conclusion:**

HIV infection causes elevated levels of C4BP and diminishes the serum levels of free protein S. We infer that the risk of thrombosis (as measured by these biomarkers) decreases with the use of antiretroviral drugs.

## Introduction

The introduction of antiretroviral treatment (ART) has dramatically improved the survival of persons living with HIV (PLWH).

However, chronic complications from the infection and from ART itself are now critical issues that confront healthcare providers managing HIV-infected patients. HIV is a chronic inflammatory state that results in the production of a variety of acute-phase-reactant proteins. Some of these have procoagulant activity. These acute-phase reactants may predispose HIV-infected patients to thrombosis.^1^

The capacity to lyse a thrombus depends not only on sufficient fibrinolytic proteins but also on the protein that activates these proteins. The most important fibrinolytic protein is plasminogen, which is normally activated by tissue plasminogen activator (tPA) to generate plasmin (the most potent protein capable of lysing thrombus). The activity of tPA on plasminogen is regulated by other proteins such as tPA inhibitor (tPAI) types I and II. Other proteins also regulate the activities of tPAI.^2^ Deficiencies in tPAI regulators often result in the unregulated activity of tPA, excessive plasminogen activation and excessive fibrinolysis. Activated protein C is an example of such tPAIs causing a deficiency of tPAI regulators and thus promoting fibrinolysis.^3^

Protein C activity is dependent on the availability of its cofactor – protein S. Protein S serves as a cofactor for the activation of protein C by thrombin and thrombomodulin.^4^ Protein S also exerts direct anticoagulant activity by binding to factors Va, VIIIa and Xa. Protein S is a soluble protein that is available in the plasma in two forms. The free form, which is available for protein C activation, constitutes about 40% of the total plasma protein S, while the remaining 60% is bound to C4b-binding protein (C4BP) in a 1:1 complex – binding to C4BP neutralises the anticoagulant activity of protein S.^4^

C4b-binding protein beta is a regulator of the complement system. It is an acute-phase reactant that serves as a carrier protein for protein S in the plasma and is elevated in inflammatory states. It prevents the excessive activity of the complement, a system activated by inflammation.^3,5,6^ Elevated C4BP levels are found in most chronic inflammatory states, such as HIV infection.^7^ Thus, if the plasma levels of C4BP rise in PLWH, a concomitant increase in the proportion of plasma protein S bound to C4BP and a reduction in the free protein S level may result. These alterations will impair the protein C activation; consequently, activated protein C (APC) is unavailable to inactivate tPAI. The resultant non-regulated activity of tPAI may result in the continuous inhibition of tPA, the failure to generate plasmin and the non-lysis of thrombi. This inability to clear thrombi may lead to a thrombogenic state.

The protein S levels were low in PLWH in several studies.^8,9^ This might partly explain the increased level of thrombotic events noticed in this group of patients.^10,11^ We surmised that this acquired deficiency might be secondary to an elevated level of C4BP. This study aimed to assess the C4BP levels in adult Nigerians living with HIV and to assess their relationship with the serum total protein S and free protein S levels.

## Methods

The antiretroviral clinic supported by the AIDS Prevention Initiative in Nigeria (APIN) at the Lagos University Teaching Hospital (LUTH), Lagos, was established in October 2004. There are over 15 000 adults enrolled in the free treatment programme, with more than 8000 on ART.

This case–control study recruited 119 PLWH and divided them into the following two groups: 61 patients previously initiated on ART for at least 12 months who had viral suppression and 58 who were ART naïve and had not started treatment. Viral suppression was defined as less than 200 copies of HIV per millilitre of blood.^12^ Fifty-nine adults who tested negative on HIV antigen screening served as controls. All the participants were recruited consecutively from the APIN clinic. Only individuals between the ages of 18 years and 65 years were recruited.

The exclusion criteria included the following: a previous or current history of venous thrombosis, current anticoagulant therapy, pregnancy, active malignancy, an AIDS-defining illness(es), deranged liver, kidney or haematological parameters, evidence of ART failure, and signs or symptoms suggestive of an ongoing systemic infection. The participants gave written informed consent and completed a self-administered questionnaire. After counselling, venous blood samples were taken using an evacuated blood tube collection system.

### Coagulation studies

Quantitative determination of the plasma free protein S levels was performed using an enzyme-linked immunosorbent assay (ELISA) kit (Helena Laboratories, Beaumont, Texas, United States [US]). It is a sandwich ELISA technique that uses antihuman protein S antibody to bind and quantify the free protein S after the plasma has been pretreated with polyethylene glycol (PEG) to precipitate the bound fraction. The citrated plasma for the protein S assay was immediately stored at –80 °C until the assay was done ≤ 2 weeks later. Protein S deficiency was defined as a plasma level of free protein S of < 60%.^13^

The C4BP beta assay used ELISA kits from Cusabio Laboratories (Wuhan, China). The ELISA kit incorporates a sandwich ELISA technique with C4BP beta-specific antibody for quantitative assay. High serum free C4BP was defined as levels of > 40.8 ng/dL (mean + 1 standard deviation [s.d.] of the healthy control group).

The euglobulin clot lysis time (ECLT) test was performed manually.^14,15^ The euglobulin fraction was precipitated from citrated platelet-poor plasma with glacial acetic acid. The precipitate was collected after centrifugation, the supernatant removed and resuspended in borate solution buffer, and clot formation initiated using 0.25 M calcium chloride. The time taken for complete clot lysis to occur was then recorded.

The CD4 cell count was analysed by semi-automated flow cytometry using the Partec CD4 counter (Sysmex Partec GmbH, Gorlitz, Germany). This was done within 4 h of collection. Based on the CD4 cell count, the HIV-infected cohort was subdivided into those with a CD4 cell count of < 350 cells/µL and those with a count of ≥ 350 cells/µL.^16,17^

### Data analysis

The study data were recorded on a Microsoft Excel spreadsheet for Windows 2010 (Microsoft Corporation, Redmond, Washington, US) and analysed using the International Business Machines Statistical Package for Social Sciences (IBM SPSS) for Windows version 20 (Armonk, New York, US). A test for normality was performed with the Kolmogorov–Smirnov test. Continuous variables were presented as the mean ± s.d., while categorical variables were presented as percentages and frequencies. The categorical data and intergroup continuous data were compared using the chi-square test and the analysis of variance (ANOVA) test, respectively. A *p*-value of ≤ 0.05 denoted statistical significance.

### Ethical considerations

The study was approved by the Health Research and Ethics Committee (HREC) of the Lagos University Teaching Hospital (approval number ADM/DCST/HREC/VOL.XVI/APP/846). Written informed consent was obtained from all the participants.

## Results

There were 178 subjects in the study. The first group comprised 61 adults living with HIV on ART and who had achieved virological suppression. The second group numbered 58 adults living with HIV but not on ART (ART naïve). The third group comprised 59 HIV-uninfected and healthy adults (Table 1). The mean CD4+ cell count of those living with HIV was 460 cells/mm^3^.

**TABLE 1:** General characteristics of subjects.

Variable				Study groups						F test	p
	Control			PLWH on HAART			Treatment-naïve PLWH				
	Mean ± s.d.	n	%	Mean ± s.d.	n	%	Mean ± s.d.	n	%
**Mean age (years)**	30.0 ± 14	-	-	33.2 ± 15	-	-	32.5 ± 16	-	-	1.732	0.18
**Sex**											
Male	-	29	49	-	22	36	-	21	36	-	-
Female	-	30	51	-	39	64	-	37	64	-	-
Total	-	59	100	-	61	100	-	58	100	-	-
**CD4 cell count**											
< 350 cells/µL	-	NA	-	-	19	-	-	30	-	-	-
≥ 350 cells/µL	-	NA	-	42	-	-	-	28	-	-	-
**ART**											
NRTI+NNRTI	-	NA	-	-	48	-	-	NA	-	-	-
NRTI+PI	-	NA	-	-	13	-	-	NA	-	-	-

PLWH, people living with HIV; s.d., standard deviation; HAART, highly active antiretroviral treatment; NA, not applicable; ART, antiretroviral treatment; NRTI, nucleotide reverse transcriptase inhibitor; NNRTI, non-nucleotide reverse transcriptase inhibitor; PI, protease inhibitor.

Forty-eight (of 61) HIV-infected patients on ART were on a combination of two nucleoside reverse transcriptase inhibitors (NRTIs) and one non-nucleoside reverse transcriptase inhibitor (NNRTI), while 13/61 patients were on regimens comprising a boosted protease inhibitor (PI) and two NRTIs (Table 1). Twenty-eight (of 61) of the patients on ART were also on trimethoprim-sulfadoxine prophylaxis for *Pneumocystis jirovecii* pneumonia. All participants had normal renal and liver function tests. The prothrombin time and the partial thromboplastin time were also normal in all subjects (Table 2).

**TABLE 2:** Haematological and biochemical parameters of the study groups.

Variable	Study groups			F test	p*
	Control	PLWH on HAART	Treatment-naïve PLWH
Haemoglobin (g/dL)	13.5	10.8	9.7	64.635	< 0.0001
WBC (×10^9^)	4.2	4.9	5.5	1.737	0.19
Platelet count (×10^9^)	289	191	194	0.31	0.86
Creatinine (mmol/L)	62.3	81.72	96.86	2.062	0.15
Alanine transaminase (mmol/L)	24.4	26.2	28.07	0.335	0.56
Prothrombin time (s)	12.44	12.80	12.81	1.720	0.18
aPTT (s)	36.65	36.06	36.39	0.731	0.48

aPTT, activated partial thromboplastin time; HAART, highly active antiretroviral treatment; PLWH, people living with HIV; WBC, white blood cell count.

* Analysis of variance test applied.

The mean plasma free protein S level of the patients on ART was significantly higher than that of the treatment-naïve but HIV-infected cohort. Conversely, there was no statistically significant difference between the protein S levels of the HIV-infected subjects on ART and the control group (Table 3).

**TABLE 3:** Protein S, C4b-binding protein beta and euglobulin clot lysis time values of the study groups.

Variable	Control	PLWH on HAART	Treatment-naïve PLWH	F test	p*
Free protein S (%)	94.9% ± 7.9%	86.9% ± 25.8%	75.7% ± 27.3%	11.13	0.005
C4BP (ng/dL)	22.4 ± 2.4	30.7 ± 2.6	36.7 ± 1.7	9.81	< 0.0001
ECLT (s)	189.5 ± 40.7	210.0 ± 61.9	241.9 ± 34.7	16.28	< 0.0001

C4BP, C4b-binding protein beta; ECLT, euglobulin clot lysis time; HAART, highly active antiretroviral treatment; PLWH, people living with HIV.

* Analysis of variance test applied.

None of the controls had serum free protein S activity at < 60%, that is, below the lower limit of normal. In contrast, 24/119 (20.2%) of the HIV-infected group had a protein S deficiency, with a mean value of 44.6% (Table 4). The difference between the proportion of those with protein S deficiency in the ART group (*n* = 9/61; 14.8%) and those in the HIV ART-naïve group (*n* = 15/58; 25.9%) was statistically significant (χ^2^ = 16.9, *p* < 0.0001).

**TABLE 4:** Proportion of protein S deficiency in the study groups.

Study group	Protein S categorisation						Total
	Protein S < 60%			Protein S ≥ 60%		
	Mean	n	%	Mean	n	%
Control	-	0	0.0	-	59	100.0	59
PLWH on HAART	45.3% ± 13.24%	9	14.8	90% ± 18.5%	52	85.2	61
HAART-naïve PLWH	44% ± 11.33%	15	25.9	88% ± 17.04%	43	74.1	58
**Total**	**44.65% ± 12.29%**	**24**	**0.0**	**89% ± 17.77%**	**154**	**-**	**178**

HAART, highly active antiretroviral treatment; PLWH, people living with HIV.

**FIGURE 1:**
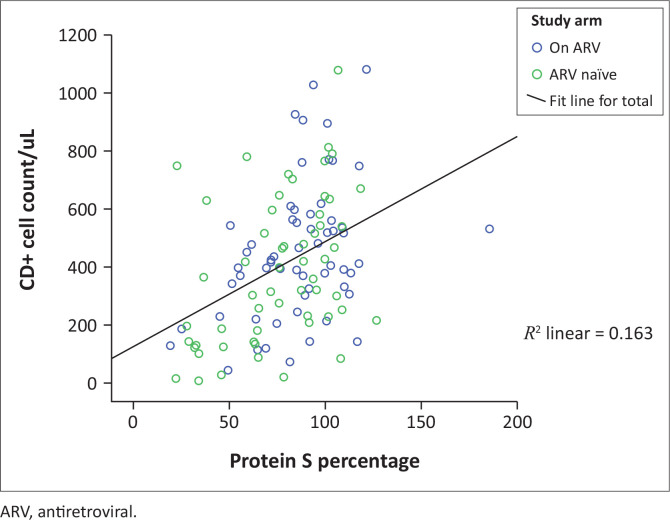
Scatterplot showing the relationship between CD4+ cell counts and serum free protein S level.

Serum free protein S deficiency (< 60%) was greater among PLWH with CD4 counts of < 350 cells/µL versus PLWH with CD4 counts of ≥ 350 cells/µL. Sixteen (32.7%) of those with CD4 counts of < 350 cells/µL had serum protein S levels of < 60% compared to 8 (11.4%) in the group with CD4 counts of ≥ 350 cells/µL (χ^2^ = 8.065, *p* = 0.005). Figure 1 confirms a positive correlation between the CD4 cell count and the protein S level of PLWH (*r* = 0.404, *p* = 0.03).

Elevated C4BP (> 40.8 ng/dL) levels were most prevalent among the treatment-naïve HIV-infected patients (*n* = 44/58; 75.9%), followed by those on ART (*n* = 26/61; 42.6%) and the controls (*n* = 11/59; 18.6%) (χ^2^ = 38.925, *p* < 0.001). The mean C4BP was significantly higher in treatment-naïve subjects living with HIV than in either those on highly active ART (HAART) or the controls (Table 3). Post hoc analysis revealed a statistically significant difference (i.e. *p* < 0.0001) between the mean C4BP levels of the PLWH on ART and those of the PLWH not on ART. There was also a statistically significant difference (i.e. *p* < 0.0001) between the mean C4BP level of the controls and that of the treatment-naïve HIV-infected patients. However, the difference between the mean C4BP level of the controls and that of the PLWH on ART did not achieve statistical significance (*p* = 0.012).

**FIGURE 2:**
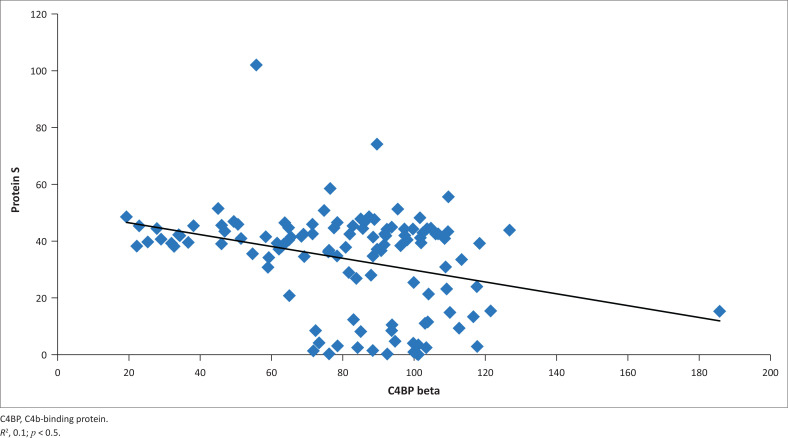
Scatterplot showing the relationship between serum levels of free protein S and C4b-binding protein.

Of the 24 PLWH who had low protein S levels, 19 (79.16%) had elevated C4BP levels, whereas of the 95 subjects living with HIV with normal protein-S levels, fewer (*n* = 51; 53.68%) had elevated C4BP levels. Protein S deficiency was more prevalent among the subjects with an elevated C4BP level (i.e. *p* = 0.02). Figure 2 confirms an inverse relationship between the C4BP and serum protein S levels.

**FIGURE 3:**
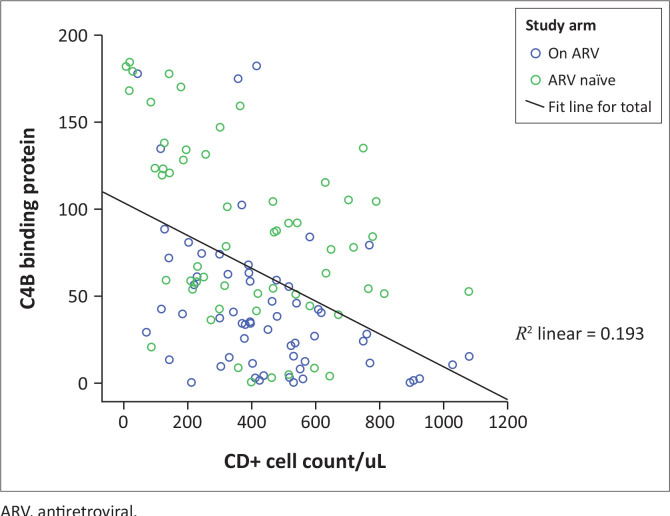
Scatterplot showing the association between CD4+ cell counts and the serum level of C4b-binding protein.

An inverse relationship (*r* = –0.439, *p* = 0.03) was noted between the CD4 cell counts of those living with HIV and their serum C4BP levels (Figure 3). A high serum level of C4BP was significantly more prevalent among those subjects with a CD4 count of < 350 cells/µL (*n* = 36/49; 73.5%) than in those with higher CD4 cell counts of ≥ 350 cells/µL (*n* = 34/70; 48.6%) (χ^2^ = 7.377, *p* = 0.008). Similarly, a negative or inverse relationship existed between the serum C4BP and serum free protein S levels (*r* = –0.539, *p* = 0.02).

The mean ECLT was significantly more prolonged among the treatment-naïve HIV-infected subjects (i.e. 241.9 ± 34.7 s) compared to the control group (i.e. 189.5 ± 40.7 s) (*p* < 0.001). However, the ECLT of those on ART (210 ± 61.9 s) did not differ significantly from that of the control group (Table 3). Post hoc comparison of the mean ECLT of the study groups showed a statistically significant difference between that of the control group and that of the cohort of treatment-naïve PLWH. Similarly, there was a statistically significant difference between the mean ECLT of those on ART and that of those infected but naïve to treatment. There was no statistically significant difference between the mean ECLT of the controls and that of those on ART (Table 5).

**TABLE 5:** Multiple comparison test of means of euglobulin clot lysis time between the study groups.

Study groups	Study group pairing	Standard error	p
Control	PLWH on HAART	9.04824	0.06
	HAART-naïve PLWH	9.20323	< 0.0001
PLWH on HAART	Control	9.04824	0.06
	HAART-naïve PLWH	9.56344	0.003
HAART-naïve PLWH	Control	9.20323	< 0.0001
	PLWH on HAART	9.56344	0.003

HAART, highly active antiretroviral treatment; PLWH, people living with HIV.

Although negligible, a correlation was demonstrated between the ECLT and the serum protein S level (*r* = –0.14, *p *= 0.04) and between the ECLT and the C4BP level (*r* = 0.16, *p* = 0.03).

## Discussion

HIV infection is associated with an increased risk of developing thrombosis. One of the proposed mechanisms to explain this increased predisposition to thrombogenesis is the derangement of procoagulant and anticoagulant substances.

This study found a significantly lower mean serum free protein S in subjects with HIV infection. The low serum free protein S level was more severe in the treatment-naïve HIV-infected subjects. Serum free protein S deficiency was noted in 20.1% of HIV-infected subjects in this study. This rate is much lower compared to the findings of Pontrelli et al.^18^ (protein S deficiency in 51% of HIV-infected children and adolescents), Bissuel et al.^10^ (protein S deficiency in 65.1% of patients with advanced HIV-1 disease) and Stahl et al.^8^ (protein S deficiency in 73% of men with long-term HIV infection). The much lower prevalence of protein S deficiency in this study may result from the exclusion of patients with symptomatic HIV illness and conditions that predispose patients to thrombosis.

Protein S deficiency was more severe in the patients who were not on antiretroviral treatment. This is similar to the pattern observed by Pontrelli et al.,^18^ who documented a decrease in the prevalence of thrombotic abnormalities in children and adolescents living with HIV once placed on antiretroviral therapy.^18^

There was a higher prevalence of protein S deficiency among HIV-infected subjects with CD4 cell counts of < 350 cells/µL. This is similar to the finding of a higher incidence of protein S deficiency among patients with CD4 cell counts of < 200 cells/µL in a study by Lijfering et al.^19^ Ahonkai et al. also reported an increased prevalence of deep venous thrombosis in HIV-infected subjects with CD4 cell counts of < 500 cells/µL.^20^ These findings suggest that continuous viral replication in treatment-naïve HIV-infected individuals may increase the risk of venous thrombosis.^21^

This study found a significantly higher level of serum C4BP in treatment-naïve HIV-infected subjects. In contrast, there was no statistically significant difference between the mean C4BP level of the HIV-infected subjects on HAART and that of the controls. Pulik et al.,^22^ in a retrospective study, reported normal levels of C4BP in HIV-infected subjects on treatment, which is similar to the findings of this study among those on HAART.

We observed an inverse relationship between the CD4 cell counts of the HIV-infected subjects and the serum C4BP level. Elevated C4BP was more prevalent in those HIV-infected persons with CD4 cell counts of < 350 cells/µL. The elevated C4BP levels support the concept that infection with HIV is a chronic inflammatory condition and the control of viral replication with the use of ART reduces the production of acute phase reactants and their attendant adverse effects.

There was a positive correlation between the serum free protein S level and the serum C4BP level. This is similar to the findings of Comp et al., who noted elevated levels of free protein S in familial C4BP deficiency.^23^ Similarly, Ceriello et al. demonstrated that low free protein S levels and activity were associated with elevated C4BP concentrations in type 1 diabetes mellitus.^24^ This finding suggests that the protein S deficiency found in HIV infection may contribute to elevated C4BP. However, this finding is contrary to that of Stahl et al.,^8^ who found no increase in C4BP in patients with low serum protein S. This disparity might be a result of the inclusion of treatment-naïve HIV-infected patients in the index study.

The mean ECLT was significantly prolonged in treatment-naïve HIV patients than in HIV patients on ART and HIV-uninfected controls. However, there was no significant difference between the ECLT of the control group and that of the HIV-infected subjects on ART. This appears to be in keeping with the findings of deranged ECLT in HIV-infected subjects who were treatment naïve by Omoregie et al.^25^ In that study, HIV infection led to a deranged fibrinolytic capacity as measured using the ECLT, thus increasing the risk of thrombotic abnormalities in HIV-infected individuals.

This study also found a negative correlation between the ECLT and serum free protein S levels. This implies that the ECLT is more prolonged when the protein S level is low and the C4BP level is high. Antiretroviral treatment significantly reduces this derangement, as shown by the absence of a statistically significant difference between the mean ECLT of healthy controls and that of HIV-infected subjects on treatment.

The limitations of this study include its cross-sectional design. A longitudinal study would provide a better long-term picture of the effects of ART on protein S deficiency and the risk of thrombosis. In addition, we used the ECLT to assess thrombophilia because it reflects the overall fibrinolytic activity of the plasma and because thrombo-elastography was unavailable at our institution.

## Conclusion

There is a reduced level of serum free protein S in HIV-infected adults. The serum level of C4BP is significantly higher in treatment-naïve HIV-infected adults than in healthy controls. There is an inverse relationship between the C4BP level and serum free protein S, suggesting that the protein S deficiency noted in this study might likely be a consequence of the elevated C4BP. The ECLT was significantly prolonged in the HIV-infected subjects compared to the healthy controls, suggesting that HIV infection leads to impaired fibrinolysis, thus predisposing individuals to thrombosis. The initiation of HAART might help to reduce protein S deficiency and the risk of thrombosis in HIV-infected individuals.
